# Potential of Overcomplete Wavelet Frame Expansion for Facilitating Electroencephalogram Information Mining

**DOI:** 10.3389/fnins.2021.782918

**Published:** 2022-01-12

**Authors:** Wanshan Liu, Xiaoyue Guo, Binqiang Chen, Wangpeng He

**Affiliations:** ^1^School of Aerospace Engineering, Xiamen University, Xiamen, China; ^2^Shenzhen Research Institute of Xiamen University, Shenzhen, China; ^3^School of Aerospace Science and Technology, Xidian University, Xi'an, China

**Keywords:** wavelet transform, EEG, brain imaging, feature extraction, overcomplete wavelet frame expansion

## Introduction

Electroencephalogram (EEG) is a record obtained by amplifying and recording the electrical activity, produced by electrical flows in the brain, from the human scalp (Zandi et al., 2011; Larson and Taulu, 2018). EEG is a widely used medium in brain imaging science and plays an important role in the research of the brain–computer interface (BCI; Gao et al., 2021). BCI is an online computerized system that converts brain signals into useful commands. To date, different types brain signals have been employed to develop BCI systems. Because of its convenience and low cost, EEG signal has become the main medium in BCI systems. However, it has been proved in practice that the acquisition of EEG signal is easily disturbed by various types of noises due to the weak energy of EEG signals. In order to extract useful information from noisy EEG signals (Shad et al., 2020), various signal processing methods are investigated in EEG signal analysis.

In the analysis of brain signals, improving the signal to noise ratio is an important preprocessing step. Traditionally, it is completed using Fast Fourier Transform (FFT) (Wahab et al., 2021). In BCI, FFT is also utilized to implement salient feature extraction from EEG signals. The short-time Fourier transform is an enhancement of FFT and it can generate two-dimensional spectral representation of EEG (Ha and Jeong, 2019). However, the main drawback of STFT is that its frequency resolution is not tunable. Huang proposed a methodology, combining STFT and convolutional neural networks for biomedical signal classification (Huang et al., 2019). In addition, the digital filters based on Fourier analysis is also an important tool for EEG signal denoising (Hsia and Kraft, 1983). Their applications include noisy artifact removal, feature selection at specific frequency bands. Although new techniques for EEG filtering are still emerging recently, the filtering technique is not important focus of BCI research. Shortcomings of digital filters are also reported in related studies (Alhammadi and Mahmoud, 2016).

During the past decades, with the increase of computing power, many more advanced signal processing methods have been invented and put into practice. Upadhyay put forward a novel technique, by integration of S transform and independent component analysis, for artifact removal and noise suppression in EEG signals (Upadhyay et al., 2016). Djemili utilized empirical mode decomposition to decompose EEG signal into intrinsic mode functions and achieved intelligent classification of normal and epileptic EEG features (Djemili et al., 2016). In the study by Jiang, a multi-dictionary based sparse representation approach is proposed for automatic detection of epileptic EEG spikes (Jiang et al., 2020). Dora applied variational mode decomposition for correcting artifacts in EEG measurements (Dora and Biswal, 2020). Chen proposed a sparse Fourier transform and applied it in power-line artifact removal (Chen et al., 2021b).

Although there are a lot of tools for EEG signal analysis, there is still a need to select practical tools to study and improve their performance. We believe that a good EEG signal processing method should have three advantages. Firstly, the method should have a rigorous mathematical basis and can be easily improved theoretically. Secondly, the method has been widely used in clinical practice, and some mature and practical technical solutions have been formed. Thirdly, the method should be computationally efficient and can be deployed quickly with conventional hardware.

## Joint of WT and Artificial Intelligence for Intelligent Analysis of EEG

Considering the above three feasible merits of a practical EEG signal analyzing tool, wavelet transform (WT) has become the most commonly used tool for EEG signal analysis. Wavelet transform is a modern development of Fourier analysis. WT can not only extract high-dimensional features from EEG, but also has high computational efficiency (Khatkar and Kumar, 2015). During the past two decades, WT has been successfully applied in EEG feature extraction and noisy reduction. Sartoretto detected features associated with epileptiform activity from EEG signals *via* discrete wavelet analysis (Sartoretto and Ermani, 1999). Mamun explored the utilization of wavelet denoising in physiological noise removal of EEG (Mamun et al., 2013). Ma proposed a method for coherence analysis, between EEG and EMG, based on wavelet decomposition (Ma et al., 2014). Asadpour designed a 4-layer Symmlet-8 wavelet transform structure for EEG signals, and effectively decomposed δ, θ, α, and β brain rhythm waves into different subspaces (Asadpour et al., 2018). Li employed wavelet packet transform (WPT) to decompose the non-stationary measurement signal in time and frequency domain, and selects the frequency band information related to the imagination task to reconstruct the EEG signal features (Li et al., 2021). Obukhov utilized ridges of wavelet spectra for automatic diagnose of epileptic seizure (Obukhov et al., 2021). It can be seen that the classical wavelet transform is mainly used to decompose EEG signals in the literature, and there is no in-depth study on the impact of wavelet transform on the decomposition results.

The wide application of wavelet transform in EEG signal analysis is not only reflected in the signal decomposition, but also in the information extraction of the decomposed subspaces. A most immediate way is to carry out statistical analysis on the decomposed wavelet subspace to obtain the corresponding statistical feature space. For example, Liu utilized many statistical indicators from selected wavelet subspaces to implement automatic seizure detection (Liu et al., 2012). In recent years, with the deepening of research, many features with physical significance have been designed and used in EEG signal classification. Zhang used sliding window technology to extract wavelet entropy, sample entropy and peak-peak value, and effectively identifies four States of driver fatigue: normal state, mild fatigue, emotional fluctuation and excessive fatigue (Zhang et al., 2014). Hadjileontiadis studied higher order spectral features in the wavelet subspaces, and proposed a novel methodology for characterization of tonic cold pain (Hadjileontiadis, 2015). Peng explored the indicator of wavelet entropy of EEG in fatigue detection, and found it provides better performance compared with FFT based indicators (Peng et al., 2021). Zarei explored nonlinear features in wavelet subspaces to improve the accuracies of automatic seizure detection (Zarei and Asl, 2021).

Although wavelet transform can reveal the time-frequency characteristics of EEG, it will take a lot of time to identify and classify them manually. This is becoming increasingly impossible in the era of medical big data. Therefore, as an important tool for feature extraction, wavelet transform also needs to be combined with artificial intelligence to achieve intelligent analysis results (Cao et al., 2019). Sharma combined wavelet subspace features and support vector machine for EEG driven epilepsy diagnosis (Sharma et al., 2020). Albaqami studied the automatic EEG signal classification using WPT and gradient boosting decision tree (Albaqami et al., 2021). Movahed employed a special orthogonal wavelet filter-bank for EEG decomposition and combined it with machine learning for diagnose the disease of major depressive disorder (Movahed et al., 2021). Shahabi studied drug responses of major depressive disorder using a technique that applied deep transfer learning on wavelet-based features from EEG (Shahabi et al., 2021). In the literature, a large number of research results show that wavelet transform is an indispensable pre-processing tool for artificial intelligence recognition of EEG signals.

## State-of-the-Art Development of Overcomplete Wavelet Frame Expansions and Its Potential Applications in EEG Analysis

The above materials show that the discrete wavelet transform is more commonly used in EEG analysis in clinical practice because of its computational efficiency. The use of wavelet transform has also been shown to be beneficial because its association with artificial intelligence can significantly improve the accuracy of clinical distortion diagnosis. Among the various types of discrete wavelet transform, classical wavelet transform and wavelet packet transform are most commonly used. They enjoy the highest computational efficiency because they are conventional linear expansions. However, both of the two signal decomposition tools have some significant shortcomings in theory. These shortcomings include translation sensitivity in wavelet subspaces (Bayram and Selesnck, 2008), fixed frequency-scale pavement (Chen et al., 2021a), and difficulties of wavelet basis information fusion.

**Figure 1:**
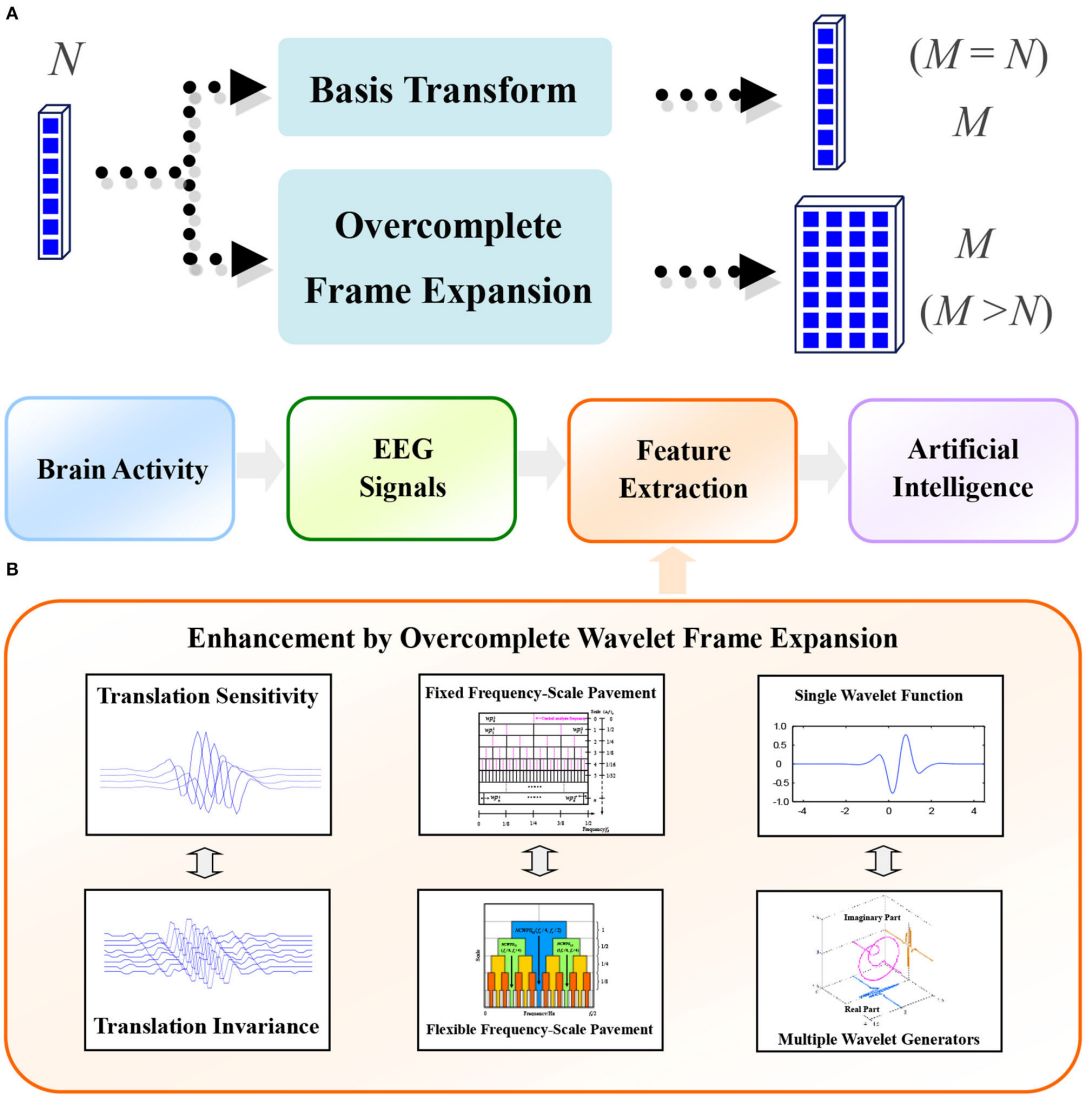
**(A)** Fundamentals of overcomplete wavelet frame expansion, where N is the number of samples in the input signal and M is the number of samples in the output signal; and **(B)** A framework to illustrate the enhancement of wavelet decomposition for EEG-based information mining.

To a large extent, the reason for these shortcomings is that the classical wavelet transform is a basis transform. For basis transformation, the number of linearly independent vectors in the basis is the same as the length of the input signal (Figure 1A). While, for overcomplete expansion, a set of linearly dependent vectors, whose number is greater than the length of the input signal, is utilized to represent the input signal (Figure 1A). This set of linearly dependent vectors is often called a dictionary. By introducing the development of frame theory, these problems can be properly resolved (Kovacevic and Chebira, 2007a,b). A framework for achieving these demands can be found in Figure 1B. Scholars have tried to improve the properties of wavelet transform from three aspects. One is to improve the translation invariance of signal expansion, the other is to adjust the “frequency-scale” pavements, and the third is to try to introduce multiple wavelet functions (Chen, 2014).

Translation sensitivity is caused by down-sampling operations in implementation of wavelet filter-bank. Although the property of perfect reconstruction can be guaranteed for the input original signal, the side effect of feature distortion in wavelet subspaces may be very serious. To address this problem, one direct and simple method is to eliminate the down-sampling operator on the filter bank, but this will significantly increase the computational complexity of the discrete wavelet analysis (Li et al., 2012). Due to the addition of redundancy, other construction constraints can be added to the construction of the wavelet bases. The constraint of Hilbert transform pair can generate approximate translation invariance with relatively small increase in calculation (Huang et al., 2021).

The property of fixed frequency-scale pavement leads to inflexible analysis results, especially when the EEG features are located in the transition region of the classical discrete wavelet passing band. Therefore, inappropriate use of classical WT and WPT will cause serious distortion of the features in the EEG decomposition results. There are two available solutions to the address this problem in the theory of overcomplete frame expansion. One is to add more wavelet functions to the wavelet base to change the basic time-frequency characteristics of the wavelet base (Selesnick, 2004). In this case, the dilation factor of the wavelet transform and the basic structure of the filter bank remain unchanged. Its disadvantage is that the effect of adjusting the frequency-scale pavement is limited, and sometimes it cannot meet the analysis requirements. The other method is to construct the wavelet time-frequency atom completely in the frequency domain by using the analytical expression (Gilles, 2013). In this case, a highly flexible dilation factor can be chosen. Its disadvantage is that there are a large number of parameters to be determined in the construction process. In recent years, some researchers have tried to use the wavelet transform with adjustable dilation factor to decompose EEG signals.

The most difficult problem is the information fusion of multiple wavelet bases with different time-frequency characteristics (He et al., 2017). With the development of sparse representation theory, wavelet bases can be used as the dictionary of signal sparse decomposition, so the information fusion becomes possible. However, the relevant decomposition models are still very limited, so they cannot be well-applied to EEG signal analysis. A more promising solution is to combine the multiple wavelet dictionaries with composite deep learning networks to achieve deep fusion of information through neural networks.

## Summary

The classical discrete wavelet transform is widely used in EEG signal analysis. The combination of wavelet transform and artificial intelligence can significantly improve the accuracy of clinical disease diagnosis. However, the influence of wavelet transform on the feature space of EEG signal decomposition is often neglected. At present, with the advancement of wavelet analysis theory, the feature space can be enhanced by using the overcomplete wavelet frame decomposition with better performance. The construction of wavelet bases and the redundancy of filter-banks can be combined with other useful construction constraints to improve the shortcomings of classical discrete wavelet transform. At present, there have been effective solutions to the property of translation sensitivity and the fixed “frequency-scale” pavement. The combination of overcomplete wavelet frame expansion and composite deep learning network can significantly promote the deep fusion of information. We recommend the use of overcomplete wavelet frame expansion in the feature extraction and signal classification of EEG, and suggest that further researches should be carried out. It should be pointed out that although overcomplete frame expansion can provide more flexible time-frequency analysis expansions, it also requires higher computational requirements. Its computation time is usually several times to tens of times that of the classical wavelet transform. Due to the increase of computing resources and the enhancement of computing power in recent years, such demands can be well-satisfied.

## Author Contributions

BC conceptualized the present work. WL, BC, and XG wrote the manuscript. WH reviewed and edited the manuscript. All authors read and approved the manuscript.

## Funding

This research was supported financially by National Natural Science Foundation of China (Grant No. 51805398), Project of Youth Talent Lift Program of Shaanxi University Association for Science and Technology (Grant No. 20200408), Fundamental Research Funds for the Central Universities (Grant No. JB211303), and Fundamental Research Funds for the Central Universities under Grant (No. 20720190009).

## Conflict of Interest

The authors declare that the research was conducted in the absence of any commercial or financial relationships that could be construed as a potential conflict of interest.

## Publisher's Note

All claims expressed in this article are solely those of the authors and do not necessarily represent those of their affiliated organizations, or those of the publisher, the editors and the reviewers. Any product that may be evaluated in this article, or claim that may be made by its manufacturer, is not guaranteed or endorsed by the publisher.
